# The relationship between preoperative psychological evaluation and compensatory sweating

**DOI:** 10.1186/s13019-018-0728-3

**Published:** 2018-05-18

**Authors:** Huai-Yu Wang, Yan-Jun Zhu, Jie Liu, Li-Wei Li, Ying-Hui Liu

**Affiliations:** 1 Thoracic Surgery Department of General Hospital of Air-Force PLA, No.30 Fucheng Road, Haidian District, Beijing, PC: 100142 China; 2 Nuclear Medicine Department of General Hospital of Air-Force PLA, No.30 Fucheng Road,Haidian District, Beijing, 100142 China

**Keywords:** Hyperhidrosis, Sympathectomy, Compensatory sweating, EPQ, Personality characteristic

## Abstract

**Background:**

This study analyzes the relationship between preoperative psychological states of primary palmar hyperhidrosis patients and postoperative compensatory sweating.

**Methods:**

We evaluated the psychological states of patients with primary palmar hyperhidrosis who received sympathectomy in our hospital from 2016 to 2017. The relationship between preoperative psychological states and postoperative compensatory sweating were assessed using Spearman’s rank-order correlation.

**Results:**

Fifty-five patients who received R4 + R3 bypass transection accepted the preoperative questionnaire survey; 35 were males and 20 were females. The average age was 24.0 ± 6.3 years (range, 14–44 years). Depression symptoms were present in 21.9% (12/55) of the patients; the incidence of anxiety was almost similar, at 23.7% (13/55). Compensatory sweating occurred in 67.3% (37/55) of the patients; of these, 56.4% (31/55) was mild and 10.9% (6/55) was moderate. None of the patients had severe compensatory sweating. There was no significant relationship between the scores of SDS, SAS, and the incidence of postoperative compensatory sweating (*P*>0.05). However, the psychoticism scale displayed a strong impact on the degree of compensatory sweating (*P*<0.05). The higher the degree of psychoticism scale, the more serious the degree of compensatory sweating.

**Conclusions:**

The results of this study showed that patients with primary palmar hyperhidrosis are more likely to have mild or moderate mental disorders, and that postoperative compensatory sweating may impact the satisfaction of surgery. In addition, the personality characteristics of patients are related to compensatory sweating.

## Background

Primary palmar hyperhidrosis (PPH) refers to a chronic disease in which hands sweat more than normal due to emotional stress. The symptoms of this kind of sweating mainly appear in childhood and adolescence [1], characterized as mild, moderate, or severe. At present, the specific pathogenesis of this disease is still unclear; however, it is mostly believed to be associated with sympathetic nervous system dysfunction caused by certain genetic factors [2]. PPH often causes embarrassment among afflicted patients; some develop serious social psychological problems, which may affect the quality of life and even lead to lifelong mental disorders [3–5].

One of the most popular treatment of PPH in recent years is endoscopic thoracic sympathectomy [6, 7]. It is used worldwide and has been proven to be safe and effective. However, compensatory sweating (CS) sometimes affects the quality of life postoperatively [8]. Current views are that the incidence of CS varies from 3 to 98%, and seems to be dependent on the height of the sympathetic chain resection. However, clinical evidence has shown that the incidence of CS varies even among patients with the same level of resection [9]. So far, no study has focused on the relationship between preoperative psychological state of the patient and CS.

In this study, we evaluated the degree of depression, anxiety, and personality characteristics of patients with severe PPH, and assessed whether these psychological factors have an impact on morbidity and postoperative satisfaction.

## Methods

This was a cross-sectional study, which included pediatric and adult patients with severe PPH who received treatment in our hospital from 2016 to 2017. Patients who were unable to complete the questionnaire independently were excluded.

All patients were assessed by the same preoperative psychologist in the same office. Each patient had to complete three copies of questionnaires independently: the Self-Rating Depression Scale (SDS), invented by Zung in 1965 [10]; Self-Rating Anxiety Scale (SAS), invented by Zung in 1971 [11]; and Eysenck Personality Questionnaire (EPQ), invented by Eysenck in 1981 [12]. The EPQ comprises 50 items to be answered in a binary manner (‘yes’/‘no’). The responses define three independent personality dimensions: (i) E dimension refers to extraversion, characterized by enjoyment of social interactions and in novel activities and a tendency towards impulsive behavior; (ii) N dimension refers to neuroticism, defined by feelings of anxiety, depressed mood, and guilt; and (iii) P dimension refers to psychoticism, defined as cold, impersonal, lacking in sympathy, emotionality, empathy, and insight combined with a tendency of paranoid ideas against oneself. These personality dimensions are complemented by a social desirability scale evaluating the tendency to respond in a manner that is assumed to be viewed favorably by others. Following completion of the questionnaires in an independent and comfortable environment, doctors would receive the feedback which was then sealed; only the researchers can utilize these personal data henceforth.

Thoracoscopic sympathectomy was performed on all patients by the same surgical team with the patient in the Semi-Fowler’s position with both arms elevated and supported to 90° under general anesthesia using a single lumen endotracheal tube. The costal pleura on the surface of the sympathetic chain were transected at the level of the fourth rib bed (R4 sympathectomy) with diathermy. In order to amputate the potential bypass nerve fibers, we extended the transection range by approximately 2 cm laterally along the surface of the corresponding rib. Then, the bypass of R3 was also transected with diathermy; however, the sympathetic chain of R3 was preserved. A temporary 10-Fr chest tube was inserted into the pleural cavity through the surgical incision and connected to a water seal system. After re-inflating the lungs, the chest tube was quickly removed and the incision was closed. We routinely conducted the operation on the right side first, in view of the imbalanced innervation of the heart by bilateral sympathetic nerves. The same procedure was performed on the contralateral site. A postoperative routine chest X-ray was obtained for all patients the following day. Most of the patients were discharged on the same day. Patients underwent follow-up 6 months after operation. The follow-up questionnaire included assessment of improvement after treatment, CS, and quality of life.

The clinical presentation of CS can be classified as mild, moderate, or severe [13]. Mild CS is sweating that occurs in small amounts, and is triggered by ambient heat, psychological stress, or physical exercise. The sweat that forms does not flow, is tolerable, and does not cause embarrassment or the need to change clothes. Moderate CS is sweating that occurs in moderate amounts, with similar triggering factors as mild CS. The sweat coalesces into droplets that flow, although not necessitating a change of clothes. Therefore, the sweating, although uncomfortable, does not embarrass the patient. Severe or intense CS is when sweating occurs in large amounts, with similar triggering factors and mild and moderate CS. The sweat droplets that form flow profusely, requiring a change of clothes one or more times a day.

All descriptive statistics are expressed as the mean ± standard deviation (SD) for continuous variables. Correlations between variables were analyzed using Spearman’s rank-order correlation. A *p* value of < 0.05 was considered to be statistically significant at the 0.05 level. Statistical analyses were performed using the SPSS statistical software version 19.0 (SPSS, Inc., Chicago, IL, USA).

## Results

A total of 55 patients accepted the preoperative questionnaire survey; 35 were males and 20 were females. The average age was 24.0 ± 6.3 years (range, 14 – 44 years). The patients’ age distribution were as follows: 56.4% (31/55) between the ages of 20 and 29 years, 9.1% (5/55) between 30 and 39 years, 29.1% (16/55) between 10 to 19 years, and 5.4% (3/55) over the age of 40 years (Table 1).

**Table 1:** Characteristics of patients with PPH and follow-up results after surgery

Variable	n	(%)
Age groups (years)		
10-19	16	(29.1)
20-29	31	(56.4)
30-39	5	(9.1)
Over 40	3	(5.4)
Gender		
Male	35	(63.6)
Female	20	(36.4)
CS		
Severe	0	0
Moderate	6	(10.9)
Mild	31	(56.4)
CS affected area		
Back	30	(54.5)
Prothorax	20	(36.4)
Thigh	7	(12.7)
Shank	3	(5.5)
Hip	4	(7.3)
Craniofacial	2	(3.6)
Axillary	1	(1.8)

*CS* compensating sweating; *PPH* primary palmar hyperhidrosis

Depression symptoms were present in 21.9% (12/55) of PPH patients; the incidence of anxiety was almost similar, at 23.7% (13/55). Almost 40% (22/55) of the patients had at least one mental disorder; 5.4% (3/55) had two. Fortunately, none exhibited severe depression or anxiety; however, the incidence of moderate and mild depression was 5.5% (3/55) and 16.4% (9/55), respectively. The prevalence of moderate and mild anxiety was 5.5% (3/55) and 18.2% (10/55), respectively (Table 2).

**Table 2:** Prevalence for depression and anxiety levels in PPH patients

Variable	n	(%)
Depression		
Severe	0	0
Moderate	3	(5.5)
Mild	9	(16.4)
Anxiety		
Severe	0	0
Moderate	3	(5.5)
Mild	10	(18.2)

*PPH* primary palmar hyperhidrosis

No patients had postoperative pneumothorax, hemothorax, Horner’s syndrome, and recurrence of palmar hyperhidrosis. CS occurred in 67.3% (37/55) of the patients; of these, 56.4% (31/55) was mild and 10.9% (6/55) was moderate. None of the patients had severe CS. The regions involved in CS were the back in 54.5% (30/55), protothorax 36.4%(20/55), thigh 12.7% (7/55), shank 5.5% (3/55), hip 7.3% (4/55), head 3.6% (2/55), and axillary 1.8% (1/55). Of the 55 patients, 43.6% (24/55) had CS in more than two regions. The degree of CS was significantly dependent on the satisfaction of the patients (*P*<0.05).

There was no significant relationship between the scores of SDS, SAS, and the incidence of postoperative CS (*P*>0.05) (Tables 3 and 4). However, there was something peculiar with regards to the EPQ scales. Although the E and N scale had no impact on the degree of CS (*P*>0.05) (Tables 5 and 6), the P scale displayed a strong impact on the degree of CS (P<0.05), the higher the degree of P scale, the more serious the degree of CS (Table 7).

**Table 3:** Relationship between CS and SDS scale

CS state	SDS			*P* value
	Mild	Moderate	Severe	
None	14	4	0	0.943
Mild	24	4	3	
Moderate	5	1	0	

*CS* compensatory sweating; *SDS* Self-Rating Depression Scale

**Table 4:** Relationship between CS and SAS scale

CS state	SAS			*P* value
	Mild	Moderate	Severe	
None	13	4	1	0.567
Mild	24	5	2	
Moderate	5	1	0	

*CS* compensatory sweating; *SAS* Self-Rating Anxiety Scale

**Table 5:** Relationship between CS and E scale of EPQ

CS state	E scale of EPQ			*P* value
	Mild	Moderate	Severe	
None	0	12	6	0.263
Mild	3	21	7	
Moderate	0	5	1	

*CS* compensatory sweating; *EPQ* Eysenck Personality Questionnaire

**Table 6:** Relationship between CS and N scale of EPQ

CS state	N scale of EPQ			*P* value
	Mild	Moderate	Severe	
None	1	14	3	0.659
Mild	2	21	8	
Moderate	0	5	1	

*CS* compensatory sweating; *EPQ* Eysenck Personality Questionnaire

**Table 7:** Relation between CS and P scale of EPQ

CS state	P scale of EPQ			*P* value
	Mild	Moderate	Severe	
None	11	6	1	< 0.05*
Mild	1	29	1	
Moderate	0	2	4	

*CS* compensatory sweating; *EPQ* Eysenck Personality Questionnaire

## Discussion

In this study, we found that the incidence of PPH had nothing to do with gender. Patients under 20-29 years old preferred to go to a doctor; this finding was consistent with the results of the studies by Fenilli, Kauffman, and Wolosker [14–16].

Endoscopic thoracic sympathectomy is currently one of the most common treatment for PPH. In this study, the cure rate of PPH was 100%. No patient complained of relapse.

While CS is a common postoperative complication among patients with PPH, its etiology is still unclear. The location in which CS occurs is also not fixed; in this study, the site of CS occurrence was widely varied, including the back (54.5%), protothorax (36.4%), thigh (12.7%), shank (5.5%), hip (7.3%), head (3.6%), and axilla (1.8%). Of the 55 patients, 43.6% had CS in more than 2 regions.

Approximately 21.9% of the patients in this study had combined mild or moderate depression, compared to the worldwide incidence of depression, which is approximately 16% [17]. According to a study in Brazil, the incidence of depression in patients with chronic disease was 15-61% [18]. In this study, 23.7% of the patients had preoperatively combined mild and moderate anxiety. In the general population, the incidence of anxiety is approximately 16%; however, when combined with other chronic diseases, the incidence of anxiety increased to 18-35% [19, 20]. The incidence of depression and anxiety among patients with PPH was slightly higher than the general population, although with a lower degree. In terms of CS, gender did not affect the incidence of depression and anxiety. Preoperative depression and anxiety did not affect the incidence of postoperative CS.

The EPQ score for PPH patients, especially the P dimension, had a certain relation with postoperative CS. While the E, and N scale scores had no obvious relation with the postoperative CS, the P scale score showed an obvious relation; the higher the P scale score, the more severe the postoperative CS. The P scale refers to psychoticism, defined as cold, impersonal, lacking in sympathy, emotionality, empathy, and insight combined with a tendency of paranoid ideas against oneself.

To some extent, there are many subjective factors in the evaluation of sweating. At present, we have not identified the etiology of CS; therefore, we could not exclude the fact that patients may have exaggerated their CS during evaluation. Compared with the SDS and SAS scales, the EPQ scale evaluates the personality characteristics of patients; these indicators can reflect the personality of patients in a more stable way. The state of depression and anxiety may be caused by the symptoms of the disease. Operations are usually associated with a quick and obvious effect, while the state of depression and anxiety may improve compared with the preoperative state. However, we should be aware that neither SDS nor SAS can represent the patients’ persistent mental state. However, personality traits are gradually formed as a person ages, and thus cannot be altered by an operation. Therefore, the EPQ scale is able to reflect personality traits’ effect on sweating more accurately. Personality traits are gradually formed as a person ages, and thus cannot be altered by an operation. Therefore, the EPQ scale is able to reflect personality traits’ effect on sweating more accurately. So we suggest that all patients with PPH should take psychological states evaluation preoperatively. And predominant high level P scale may be considered as a relative contraindication for endoscopic thoracic sympathectomy. For these patients, we should recommend medication therapy, such as oxybutynin as first line of treatment [16].

## Conclusions

The results of this study showed that patients with PPH are more likely to have mild or moderate mental disorder, and that postoperative CS may impact the satisfaction of surgery. In addition, the personality characteristics of patients are related to CS. All patients with PPH should take psychological states evaluation preoperatively. And certain psychic characteristics may be considered as a relative contraindication for surgery.

## Abbreviations

CS: Compensatory sweating
EPQ: Eysenck Personality Questionnaire
PPH: Primary palmar hyperhidrosis
SAS: Self-Rating Anxiety Scale
SDS: Self-Rating Depression Scale
